# Health Consciousness, Sensory Appeal, and Perception of Front-of-Package Food Labels as Predictors of Purchase Intention for Unhealthy Foods in Peruvian University Students

**DOI:** 10.3390/nu17111921

**Published:** 2025-06-03

**Authors:** Jacksaint Saintila, Rafael Orlando Florián-Castro, Eufemio Magno Macedo-Barrera, Raquel Patricia Pérez-Facundo, Yaquelin E. Calizaya-Milla

**Affiliations:** 1 Research Group for Nutrition and Healthy Behaviors, Universidad Señor de Sipán, Lambayeque 14001, Peru; 2 Escuela de Posgrado, Universidad San Ignacio de Loyola, Lima 15024, Peru; rafael.florian@usil.pe; 3 Facultad de Bromatología y Nutrición, Universidad Nacional José Faustino Sánchez Carrión, Huacho 15135, Peru; emacedo@unjfsc.edu.pe; 4 Área de Diseño y Desarrollo, Bakels Peru SAC, Lima 150103, Peru; ratchel835@gmail.com; 5 Research Group for Nutrition and Lifestyle, Universidad Peruana Unión, Lima 15464, Peru; yaquelincalizaya@upeu.edu.pe

**Keywords:** convenience food, consumer behavior, feeding behavior, food preferences, nutritional labeling, university students, Peru

## Abstract

**Background:** Health consciousness refers to an individual’s level of knowledge and concern regarding the impact of food on personal health; sensory appeal to the influence of attributes such as taste, aroma, appearance, and texture on food preference; and perception of front-of-package (FOP) labels refers to how the presentation of nutritional information on the package affects product choice. Given the increasing concerns about unhealthy food consumption among university students and the role of FOP labels in guiding food choices, it is essential to understand how these factors influence purchase intentions. **Objective:** This study was to examine the relationship between health consciousness, sensory appeal, and perception of FOP labels with purchase intentions for unhealthy foods in university students. **Methods:** A cross-sectional predictive study involved 361 students from public and private universities using a non-probability purpose-sampling approach. Data were collected through a previously validated questionnaire and analyzed using multiple linear regression. Results: The results revealed a significant positive association between sensory appeal and purchase intentions for unhealthy foods (β = 0.339; *p* < 0.001). In contrast, health consciousness (β = −0.296; *p* < 0.001) and perception of FOP labels (β = −0.237; *p* < 0.001) were inversely related to purchase intentions. **Conclusion:** These findings suggest that promoting health consciousness, improving perceptions of FOP labels, and addressing sensory appeal could effectively encourage healthier eating habits and prevent diet-related diseases among university students.

## 1. Introduction

**Figure 1:**
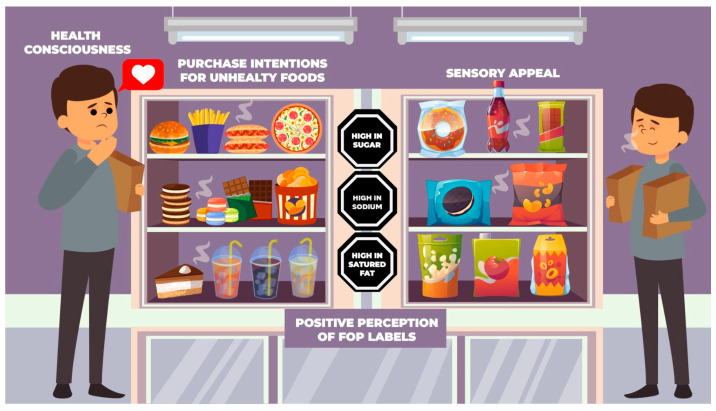
What Drives University Students to Choose Unhealthy Foods? Note. FOP: front-of-package. The central role of university students in the intention to purchase unhealthy foods is symbolized by the figure of the student approaching a store that offers unhealthy food options.

The intention to purchase unhealthy foods refers to an individual’s inclination to buy food that has low nutritional value [1]. This intention is an important indicator of understanding and potentially changing food consumption patterns. Purchase intentions for unhealthy foods among university students can be influenced by multiple factors, including health consciousness, sensory appeal, and perception of FOP food labels (Figure 1) [2,3,4,5].

In Peru, as in many countries, several studies have examined the eating habits of university students, revealing a worrying trend toward the consumption of unhealthy foods [6,7,8,9,10]. At the university stage, students often opt for food choices that are convenient but nutritionally deficient [6]. Due to factors such as lack of time, academic stress, and limited availability of healthy options in the university environment, students often consume fast foods and snacks that are high in saturated fats, added sugars, and sodium [9]. In addition, academic and social pressures, along with a lack of cooking skills, contribute significantly to these unhealthy dietary choices [8]. These dietary patterns are concerning because they can establish unhealthy long-term lifestyle habits, increasing the risk of non-communicable diseases such as obesity, type 2 diabetes, and cardiovascular disease [7].

According to data from the National Institute of Statistics (INEI), in 2022, 37.5% and 25.6% of people aged 15 years and older in Peru will be overweight and obese, respectively [11]. Obesity affects urban areas and women the most, with a prevalence of 39.0% and 29.8%, respectively [11]. The prevalence of diabetes is 8.1% among women and 7.2% among men, with approximately two new cases per 100 people annually [12]. In 2021, 17.2% of the Peruvian population over the age of 15 had high blood pressure [13]. According to INEI estimates, 39.9% of Peruvians aged 15 years and older have at least one comorbidity [14]. Furthermore, a report estimates that 16% of the Peruvian population over the age of 20 suffers from cardiovascular disease, and more than 22,000 people die from some form of heart failure [15]. Unhealthy eating behaviors, characterized, for example, by low consumption of fruits and vegetables, increase the probability of developing these diseases [7]. In Peru, only 11.3% of adults consume the recommended daily amount of fruits and vegetables, which is 400 g/day or 5 servings/day [13].

Health consciousness refers to the awareness and concern individuals have about how their diet affects their health [4]. Among university students, this awareness can influence food choices, particularly as they begin to assume greater responsibility for their eating habits [16]. Previous research has shown that greater awareness of personal health and increased concern for well-being are related to better dietary choices [17]. According to Ellison et al. [18], individuals with a high level of health consciousness are more likely to read nutrition labels carefully and purchase low-calorie foods. This trend is supported by a study that found that health-conscious individuals are 3.2 percentage points more likely to consume the recommended daily intake of fruits and vegetables (i.e., five servings per day) compared to their less health-conscious counterparts. Moreover, they are 18.8 percentage points more likely to consume three to four servings per day, which, while slightly below the recommended intake, still reflects a substantially healthier consumption pattern [19]. Another study has shown that health consciousness has a significant impact on purchasing decisions, leading consumers to choose healthier alternatives [20]. This is supported by Vainio et al. [21], who emphasize the link between health consciousness and the adoption of healthier diets. While health consciousness has been associated with healthier food choices in the general population, there is limited evidence on whether this relationship holds true specifically among university students, who often engage in unhealthy eating behaviors.

Sensory appeal—encompassing taste, smell, texture, and appearance—plays a key role in food preferences and can often outweigh nutritional considerations, especially among young consumers [22]. The sensory appeal to foods is closely related to dietary decisions, with a significant impact on the selection of unhealthy foods [23]. Sensory attributes of foods, such as sweet taste or pleasant texture, are key determinants of food preferences, especially in university students, leading them to choose less healthy options [5]. Drewnowski and Almiron-Roig highlight that individuals often prefer foods with intense flavors and satisfying textures, leading them to choose calorie-dense options [24]. In addition, Robinson et al. [25] report that sensory attributes, such as visual presentation and aroma, have a stronger impact on adolescents’ food preferences and consumption decisions than nutritional knowledge. These findings emphasize the importance of taking sensory appeal into account when designing nutritional interventions to improve the habits of young people, recognizing that sensory satisfaction plays a crucial role in their food choices.

Front-of-pack (FOP) food labels are designed to help consumers make healthier food choices by providing accessible nutritional information at the point of purchase [26,27,28]. Research has shown that clear and prominent labels, such as warning symbols or color-coded systems, can influence perception and reduce purchase intention for unhealthy products [29]. In Peru, the implementation of black octagonal FOP warnings offers a unique opportunity to study how these labels affect young adults’ food decisions, particularly in a university environment where unhealthy options are prevalent [30]. FOP labels can discourage consumers from purchasing unhealthy products and reduce the perception that these products are healthy [26,27,28]. Furthermore, FOP labels that clearly highlight the unhealthy content of products can reduce their appeal and decrease purchase intent [31]. Recent systematic reviews of experimental and quasi-experimental studies support the view that FOP labels lead to a reduction in unhealthy product choices of between 26% and 36% [28]. Additionally, a study carried out in Chile, one of the leading countries to implement mandatory FOP warnings, found that FOP labels were linked to a 24% decrease in the purchase of unhealthy foods [27]. Therefore, front-of-pack labeling can inform consumers and act as an effective deterrent against unhealthy food choices.

**Figure 2:**
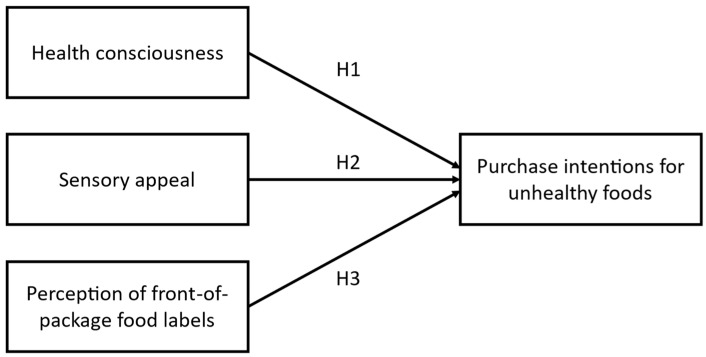
Hypothesis of the study. Note. H1: Health consciousness is negatively associated with the purchase intentions for unhealthy foods; H2. Sensory appeal is positively correlated with purchase intentions for unhealthy foods; H3. Perception of front-of-pack food labels is negatively associated with the purchase intentions for unhealthy foods.

Although previous studies have examined the influence of health consciousness, sensory appeal, and FOP label perception on food choices in general populations, little is known about how these factors interact specifically among university students. This age group is exposed to unique stressors and eating environments that may alter their responses to health cues and labeling interventions. In Peru, where FOP warning labels have been recently implemented, and unhealthy food consumption remains high, understanding these relationships is particularly relevant. This study addresses this gap by analyzing psychological and informational predictors of unhealthy food purchase intention in Peruvian university students, which is especially important for public health measures and nutritional interventions on college campuses. Therefore, the purpose of the study was to examine the relationship between health consciousness, sensory appeal, and perception of FOP labels with purchase intentions for unhealthy foods in university students. In addition, Figure 2 presents the following hypotheses:

## 2. Materials and Methods

### 2.1. Design and Participants

A quantitative cross-sectional predictive design study was conducted to explore the influence of health consciousness, sensory appeal, and perception of FOP food labels (predictor variables) on the intention to purchase unhealthy food (criterion variable) among students from public and private universities in Peru.

Participants were selected by nonprobability convenience sampling [32,33]. The sample size was calculated using the Soper Free Statistics Calculators [34]. The multiple linear regression analysis incorporated three explanatory variables. For an effect size of 0.10, a statistical power set at 0.90, and a significance level (α) of 0.05, the analysis required a minimum sample size of 145 participants. Data were collected using an online survey questionnaire between October and November 2024. Participants were recruited from one public and one private university, both located on the North coast of Peru. However, these institutions have a diverse student population, with individuals coming from various regions of Peru to pursue higher education. The recruitment process was conducted in coordination with university professors who served as administrators of student WhatsApp groups. After obtaining their approval, we were granted access to these groups, where we formally invited students to participate in the study. This strategy allowed us to reach a diverse group of students enrolled in different academic programs. The survey was developed and administered through the QuestionPro Survey Platform.

The study included undergraduate students who were enrolled in the 2024-II academic semester at the selected universities. Regular students (those taking more than 12 academic credits, as defined by the Peruvian educational system) were eligible to participate. Additionally, only students who voluntarily accepted the informed consent and fully completed all study variables were included.

Exclusion criteria included students who did not fully complete the questionnaire, as incomplete responses could bias the analysis. Additionally, international (foreign) students were excluded to maintain a more homogeneous sample, considering potential differences in dietary habits, cultural influences, and food purchasing behaviors compared to local students.

Therefore, a total of 361 university students were considered in this study. The majority of the participants (58.2%) were females. Additionally, 65.4% of the students studied disciplines other than health sciences. Table 1 presents the sociodemographic characteristics of the participants.

### 2.2. Ethical Aspects

The study protocol was reviewed and approved by the Research Ethics Committee of the Faculty of Sciences of the Universidad Señor de Sipán (FCS-USS-0023–2024). Participants were informed of the purpose of the study, the voluntary nature of their participation, and the confidentiality of the information collected. Written informed consent was obtained from all participants. Data collection was carried out in accordance with the ethical criteria defined in the Declaration of Helsinki and its subsequent amendments.

### 2.3. Variables

Health consciousness. In the current study, participants’ health consciousness of participants was assessed using a 3-item scale [35,36]. The items were designed on a 7-point Likert-type scale (7 = strongly agree and 1 = strongly disagree). A sample item on this scale is “I chose food carefully to ensure good health”. The scale demonstrated good reliability in our study, with a Cronbach alpha of 0.71. The total score for health consciousness was calculated as the sum of the three items, with higher scores indicating greater health consciousness.

Sensory appeal. Sensory appeal was measured using a three-item scale [37]. The items were designed on a 7-point Likert-type scale (7 = strongly agree and 1 = strongly disagree) [38,39]. An example item of this scale is “Suboptimal food has a pleasant texture”. The scale showed moderate reliability in our study, with a Cronbach alpha of 0.69. The overall sensory appeal score was obtained by summing the responses to the three items, with higher scores reflecting stronger sensory motivation.

**Figure 3:**
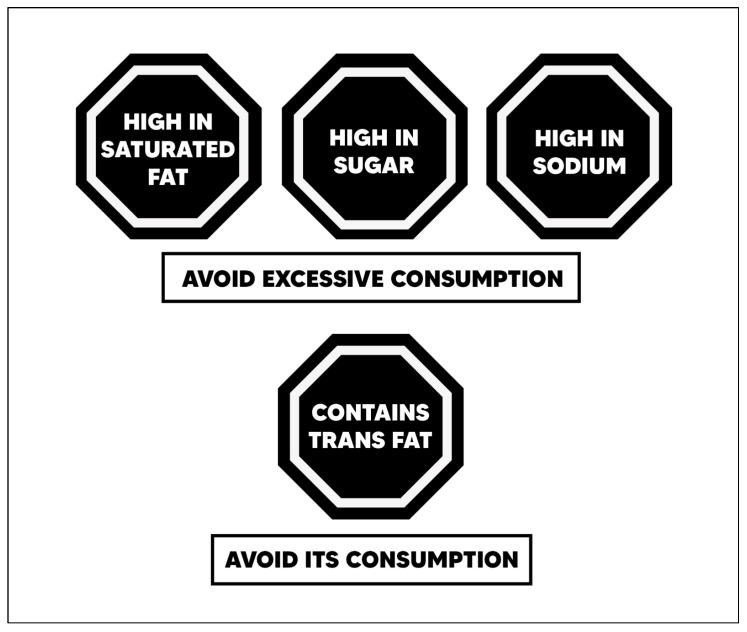
Octagonal Front-of-Package Warning Labels Implemented in Peru’s Food Labeling Policy.

Perception of front-of-pack food labels. The four-item scale used in this study to assess the perception of FOP labels for food was adapted from an instrument created and validated in previous studies, where the relevant literature was used to select the included items [40,41]. Items were measured using a 7-point Likert scale, where 7 indicates a positive opinion (strongly agree), and 1 represents a negative opinion (strongly disagree). An example item is: “I am satisfied with the use of octagons in Peru”. The scale showed good reliability in our study, with a Cronbach alpha of 0.74. The perception of FOP labels score was calculated by summing the four items, with higher scores reflecting a more favorable perception of warning labels. To ensure that all participants evaluated the same visual stimulus, the online survey included images of Peru’s official octagonal warning labels. These labels, mandated by national food regulations, feature warnings such as “High in Sugar”, “High in Saturated Fats”, “High in Sodium”, and “Contains Trans Fats” [42]. The images were displayed before participants responded to the scale items to standardize their interpretation of FOP warnings. A sample of these labels is provided in Figure 3.

Purchase intentions for unhealthy foods. Purchase intentions for the unhealthy foods of the participants were evaluated with a 4-item scale [36,43]. These items were designed on a seven-point scale (7 = strongly agree and 1 = strongly disagree) [38]. For example, one question is, “I am willing to consume suboptimal foods if they are available for purchase. The alpha reliability measured for this scale in our study was 0.82. The total score was calculated as the sum of the four items, with higher scores indicating stronger purchase intention for unhealthy foods. The full instrument can be found in Supplementary Materials.

To ensure clarity in participants’ understanding of “unhealthy foods”, we provided examples within the survey, including sugary beverages, processed snacks (such as chips and cookies), fast food (e.g., hamburgers and fried chicken), and ultra-processed packaged foods. These examples were included to standardize interpretations and minimize variability in how respondents perceived the concept of unhealthy foods. Additionally, since the survey was conducted online, we incorporated illustrative images of these food categories to further standardize interpretations and minimize variability in how respondents perceived the concept of unhealthy foods.

Before data collection, the questionnaire was pretested with a small group of university students (n = 26) to assess clarity, comprehensibility, and completion time. Based on participant feedback, minor modifications were made to improve the wording of certain items and ensure ease of understanding.

### 2.4. Statistical Analysis

Sociodemographic variables were analyzed using tables with absolute frequencies and percentages. To assess the distribution of the variables, the Shapiro–Wilk test was performed. Since the results indicated that the variables did not follow a normal distribution (*p* < 0.05), the non-parametric Mann-Whitney U test was used to examine the difference in variables of interest according to the gender of the participants. Similarly, the Spearman correlation was used to assess the correlation between the variables under study. Regarding predictive analysis, the multiple linear regression method was used to examine the relationship between predictor variables health consciousness, sensory appeal, and perception of FOP labels with the purchase intention of unhealthy foods (criterion variable). A significance level of 5% was used to ensure the reliability and validity of the results obtained. Data processing and analysis were performed using SPSS version 25 statistical software (SPSS Inc., Chicago, IL, USA).

## 3. Results

When comparing scores by sex, no statistically significant differences were observed in sensory appeal (U = 17,204.50, *p* = 0.505), health consciousness (U = 16,009.50, *p* = 0.073), perception of front-of-pack (FOP) labels (U = 16,009.50, *p* = 0.074), or purchase intention for unhealthy foods (U = 16,935.00, *p* = 0.358). However, female students showed slightly higher median scores in purchase intention (16.0 vs. 15.0) and slightly lower scores in perception of FOP labels (20.0 vs. 21.0) compared to male students (Table 2).

Table 3 shows the correlations between the variables under study. Greater sensory appeal is associated with greater purchase intention for unhealthy foods (*rho* = 0.346, *p* < 0.01). In addition, health consciousness (rho = −0.261, *p* < 0.01) and perception of FOP labels (rho = −0.221, *p* < 0.01) are negatively correlated with purchase intention for unhealthy foods.

The regression analysis showed that the determination coefficient R^2^ = 0.27 indicates that the predictor variables health consciousness, sensory appeal, and the labels of FOP foods explain 27% of the variability in the purchase intention for unhealthy foods. The F-value of ANOVA (F = 43.10, *p* < 0.001) indicates that there is a significant linear relationship between the predictor variables (health consciousness, sensory appeal, and perception of the FOP food labels) and the criterion variable (purchase intention for unhealthy foods).

The βs coefficients are shown in Table 4. It is evident that the predictor variables (health consciousness, sensory appeal, and perception of the FOP labels) significantly predict the purchase intention for unhealthy foods (criterion variable). The t-value of the regression beta coefficients of the predictor variables is highly significant (*p* < 0.001). Similarly, the βs coefficients indicate that the sensory appeal variable has the highest weight in the predictors (0.339), followed by health consciousness (−0.296) and perception of the FOP labels (−0.237). Regarding the sociodemographic variables, the standardized coefficient for sex (β = −0.190, *p* < 0.001) suggests that women are less likely to purchase unhealthy foods compared to men. Likewise, the discipline of studies exhibits a significant negative relationship with purchase intention (β = −0.250, *p* < 0.001), indicating that individuals studying health-related fields are less inclined to purchase unhealthy foods.

## 4. Discussion

This study explored health consciousness, sensory appeal, and perception of FOP labels as possible predictors of purchase intention for unhealthy foods in university students. One of the most notable findings is the positive and significant correlation between the sensory appeal of unhealthy foods and the purchase intention of these products. On the other hand, the results also show an inverse and significant relationship between health consciousness and the perception of FOP labels with the purchase intention for unhealthy foods.

Sensory attributes of foods play an important role in orientation to specific food sources, guiding preferences, serving selection, and the experience of satiety after consumption, and are even fundamental in the process of acquiring nutritional knowledge [23]. The results obtained in the current study indicate a positive correlation between sensory appeal and purchase intention for unhealthy foods, suggesting that as levels of sensory appeal increase, a corresponding increase in purchase intention for unhealthy foods is observed among the Peruvian university students evaluated. This finding is consistent with previous research that indicates that sensory attributes of food, such as taste, smell, appearance, and texture, have a significant impact on food purchase and consumption decisions, especially for foods that are considered unhealthy [24,25,44]. Previous studies in academic settings have shown that the appealing sensory attributes of these foods can trigger emotional responses and increase the desire to consume unhealthy foods despite the knowledge of their potential adverse health effects [5].

The importance of sensory appeal can be understood through various psychological and physiological mechanisms. For example, foods that are high in fat, sugar, or sodium often possess highly attractive sensory qualities that can activate the brain reward center, producing an immediate sense of pleasure and satisfaction. These sensory inputs are then associated with economic valuations that influence eating behavior [22,45]. This phenomenon is especially significant in the university setting, where stressors, the desire for immediate gratification, and social pressures can amplify the inclination toward foods that provide immediate sensory pleasure [46]. Therefore, these findings indicate the need to implement strategies, such as developing educational campaigns, to increase the awareness of university students of how their sensory responses influence their purchasing decisions.

Similarly, the current study has revealed a significant inverse association between health consciousness and the purchase intention of unhealthy foods, demonstrating that an increase in health consciousness is associated with a decrease in the purchase intention of unhealthy foods. Other studies have also shown that consumers’ purchasing decisions can be significantly influenced by concerns about the health and nutritional aspects of foods [4,47]. In particular, university students who are more aware of the importance of a balanced and healthy diet are more likely to consume the recommended daily amount of fruits and vegetables [19]. Similarly, people who are highly aware of the importance of a balanced and healthy diet are more resistant to the temptation of unhealthy foods; they instead choose more nutritious alternatives that benefit their well-being [18,48]. This could be because greater health consciousness may encourage a more critical and reflective approach to dietary decision-making, allowing individuals to more effectively weigh the long-term consequences of their dietary choices [3,49]. Therefore, these findings emphasize the importance of educational interventions, such as nutrition education workshops, that clearly highlight the nutritional qualities of foods and promote healthy eating habits. This would improve people’s ability to make informed decisions about their food choices, which is crucial to preventing excessive consumption of unhealthy foods.

FOP labels are designed to be easily interpreted, allowing consumers to quickly identify food products that are more beneficial to their health or those that should be consumed in moderation due to their less favorable nutritional content [26]. The ability to evaluate nutrition quickly and efficiently is particularly valuable in a university setting, where fast food is widely available and healthy options are scarce [50]. In the current study, the results also show an inverse and significant relationship between the perception of FOP labels and the intention of purchasing unhealthy foods. This is consistent with previous studies that have highlighted how FOP labels can be an effective tool for guiding consumers toward healthier dietary choices [51]. The inclusion of clear and comprehensible FOP labels can significantly improve the probability of individuals selecting foods with a superior nutritional profile [31]. Furthermore, they help raise awareness of specific nutritional aspects of foods, such as sugar, saturated fat, and sodium content, which are critical to preventing non-communicable diseases related to diet [26,27,28]. The study’s findings support the importance of enhancing FOP label initiatives, which should be based on solid scientific evidence and easily comprehensible to guide consumers in making informed food choices. It is also recommended that these measures be supplemented with educational programs to allow people to make informed and healthy food choices in different purchasing contexts [52].

Additionally, the results of the multiple regression model indicate that certain sociodemographic variables significantly influence the intention to purchase unhealthy food. For example, the findings reveal a significant negative relationship between being a woman and the intention to purchase unhealthy food. This suggests that women are less likely to purchase these products compared to men. This finding aligns with previous studies suggesting that women are generally more aware of healthy eating and prioritize more nutritious food choices [53]. In the Peruvian context, this trend may be influenced by traditional gender roles, in which women typically bear the primary responsibility for selecting and preparing food at home, thereby promoting healthier choices [54]. Furthermore, the increasing exposure to public health campaigns and educational programs highlighting the importance of a balanced diet may have had a greater impact on women, reinforcing behaviors geared toward healthier food choices [53]. The perceived responsibility for family well-being may motivate women to avoid purchasing foods considered unhealthy [55].

On the other hand, studying health sciences was significantly and negatively associated with the intention to purchase unhealthy food. This suggests that participants with a background in health sciences are less likely to purchase these products compared to those from other disciplines. This finding may be attributed to their greater knowledge of nutrition and health, which could influence their purchasing decisions and preference for healthier food options [56]. However, it is also important to consider the potential role of selection bias. Students who choose health-related fields may already be more health-conscious before entering their academic programs [56,57], which could partly explain their lower purchase intentions for unhealthy foods. While education in health sciences likely reinforces these behaviors, further research is needed to determine whether these differences are primarily due to pre-existing health awareness or the influence of formal training in health-related disciplines. In any case, these findings underscore the importance of considering sociodemographic variables in the development of strategies to promote healthy eating and public policies aimed at reducing the consumption of unhealthy food, both among university students and the general population.

In addition to the main results mentioned above, it is important to note that the predictor variables of health consciousness, sensory appeal, and perception of FOP labels account for 27% of the variability in the purchase intention for unhealthy foods. This suggests that while significant, these variables do not fully explain all possible influences on the purchase intention for unhealthy foods. This finding indicates that other factors may also have a significant impact on dietary decisions, including the social and family environment, marketing and advertising campaigns, food prices and accessibility, as well as individual psychological factors such as emotional state and pre-existing dietary habits. Additionally, this result highlights the importance of ongoing research and the development of further theoretical models that can encompass a wider range of variables. Therefore, future research should investigate the interaction between these predictors and how additional contextual and psychological factors contribute to the purchase intention for unhealthy foods. A better understanding of these mechanisms would facilitate the design of targeted public interventions and health policies to combat the excessive consumption of unhealthy foods and address the growing problem of obesity and diet-related diseases in the university setting.

Our findings, which underscore the significant influence of FOP label perception on intentions to purchase unhealthy foods, align with evidence from countries such as Chile and Mexico. For instance, one study reported that 24% of consumers reduced their purchase of foods with warning labels compared to the period before the law was enacted [27]. Another analysis indicated that Chilean households reduced their purchases of sugar by 37%, sodium by 22%, saturated fats by 16%, and total calories by 23% in products with warning labels [58]. On the other hand, one study projected that this labeling could prevent 1.3 million new cases of obesity and save $1.8 billion in healthcare costs [59]. This suggests that the implementation of clear and visible warning labels can be an effective strategy for guiding consumers toward healthier food choices. Moreover, the experience of these countries demonstrates that such policies not only influence purchasing decisions but also incentivize the food industry to reformulate their products to avoid warning labels, thereby contributing to a healthier food supply.

It is also important to consider that the findings of this study may not be equally generalizable across different geographic regions or socioeconomic contexts. Cultural norms, food availability, and marketing exposure can vary widely between urban and rural areas, potentially influencing both health consciousness and the perception of food labeling [60]. Similarly, individuals from lower socioeconomic backgrounds may prioritize affordability and accessibility over nutritional value, which can reduce the impact of FOP labels and health education strategies on food choices [61]. Studies have shown that socioeconomic disparities can affect not only dietary behaviors but also the cognitive resources available for making health-conscious decisions, especially in food environments saturated with unhealthy options [62]. Therefore, future interventions and public policies should be sensitive to these differences and adapt strategies to account for the diverse contexts in which food choices are made.

*Public health and practical implications*

The findings of this study have several important implications for both public health and practical applications. For example, the association between health consciousness and purchase intentions for unhealthy foods underscores the need to strengthen nutrition education campaigns. Public health authorities can use this information to design interventions that increase awareness of the negative effects of unhealthy foods, especially in educational settings such as universities, where the availability of healthy options may be limited. In addition, the positive perception of FOP labels as a tool to guide purchasing decisions suggests that expanding and strengthening the use of these labels could be an effective strategy to reduce the consumption of unhealthy foods in the population. Policies that promote clear and visible labeling can significantly contribute to reducing non-communicable diseases associated with unhealthy diets.

Furthermore, the strong influence of sensory appeal on purchase intentions for unhealthy foods highlights the need to integrate sensory-based strategies into health promotion efforts. University food service providers, in particular, could play a key role in this by enhancing the sensory appeal of healthier food options on campus. Research suggests that students are more likely to choose nutritious foods when they are presented in an attractive manner and prepared with appealing flavors and textures. Enhancing the visual presentation of healthy meals by incorporating vibrant colors, using sensory-based marketing techniques with descriptive menu labeling, and improving flavor profiles with herbs, spices, and alternative cooking techniques can help shift food preferences toward healthier choices.

To implement these interventions practically on university campuses, institutions could train kitchen staff and food service personnel on sensory optimization techniques and menu engineering. Establishing collaborations with nutritionists and culinary experts may help design meals that balance nutrition with sensory satisfaction. Moreover, universities could conduct regular taste-testing events and student feedback sessions to refine offerings, alongside introducing behavioral nudges such as strategic food placement and signage to promote healthier selections. Adjusting portioning and pricing strategies to make nutritious options more accessible and appealing would also contribute to a healthier food environment on campus.

*Limitations and Future Perspectives*

This study has several limitations that must be considered when interpreting the results. The use of nonprobability sampling limits the representativeness of the sample and, therefore, the ability to generalize findings to larger populations. Although the study was conducted in two universities located on the North coast of Peru, these institutions attract students from various regions of Peru. Second, relying on self-report measures introduces the possibility of biases, such as social desirability bias and recall errors. This raises questions about the accuracy of reflecting the participants’ actual intentions and behaviors in relation to their eating. Third, the cross-sectional design of the study prevents the determination of causal relationships between the variables examined. Although significant associations were identified, it cannot be ruled out with certainty that health consciousness, sensory appeal, and perception of FOP labels are direct causes of the purchase intention for unhealthy foods. Fourthly, this study did not explore the role of possible modulating effects that could influence the relationships between the variables of interest. The omission of these moderating effects may limit our understanding of how different factors interact in various contexts.

Fifthly, the reliability coefficient of the Sensory Appeal scale (Cronbach’s alpha = 0.69), although slightly below the conventional threshold of 0.70, falls within an acceptable range for exploratory research. While this value is considered moderate and acceptable in exploratory studies [63,64], it suggests that the internal consistency of this scale could be improved in future research. Potential reasons for this slightly lower reliability may include the multifaceted nature of sensory appeal and the variability in individual perceptions of food attributes. Future studies could refine this scale by testing additional items or applying alternative reliability assessments, such as composite reliability, to ensure a more robust measurement of the construct.

Furthermore, the study was conducted between October and November 2024. This period coincides with key academic demands, including midterms and final evaluations, which may contribute to higher levels of stress and time constraints among students. Research suggests that academic stress can influence dietary choices, often leading to increased consumption of convenience foods or unhealthy snacks [65]. Therefore, the results should be interpreted considering the potential impact of academic workload on students’ food purchase intentions. Future studies could explore seasonal variations in food choices to assess whether purchase intentions fluctuate across different academic periods.

Finally, another limitation to consider is the potential for selection bias among students from health-related disciplines. Students who choose to study health sciences might already possess greater health consciousness and nutritional awareness before entering their programs, influencing their lower purchase intentions for unhealthy foods. This pre-existing health orientation could partially account for the observed associations. Future research should explore longitudinal designs to determine whether these attitudes and behaviors are a result of educational experiences or inherent predispositions toward health-related behaviors.

Given these limitations, there are several promising directions for future research. First, to improve the representativeness of the sample and the generalizability of the results, it would be beneficial to use probability sampling methods. Future research could benefit from broadening the sample, geographic, and demographic scope of the study to ensure a greater diversity of participants. Longitudinal designs can help examine causal relationships between health consciousness, sensory appeal, perception of FOP labels, and purchase intention for unhealthy foods, providing deeper insights into the dynamics of these factors over time. Finally, future studies could enrich our understanding of the conditions under which variables of interest have a stronger or weaker impact on the intention to purchase unhealthy foods by incorporating the analysis of moderating effects. This could include factors such as cultural context and academic stress, among others, offering a more detailed perspective on dietary decisions in university populations.

## 5. Conclusions

This study focused on exploring how health consciousness, sensory appeal, and perception of FOP food labels are associated with the purchase intention of unhealthy foods. The results obtained support the hypotheses, indicating a positive and significant correlation between sensory appeal and the intention to buy unhealthy foods. Furthermore, an inverse and significant relationship was observed between health consciousness and perception of FOP labels with the purchase intention of these products. This study emphasizes the impact of sensory appeal on food preferences, indicating that sensory stimuli can take precedence over health concerns in the decision-making process. Furthermore, the study confirms that health consciousness and a positive perception of FOP labels are important protective factors that can discourage the purchase of unhealthy foods. These findings are particularly relevant for public health strategies and nutrition education, as they provide clear avenues for implementation.

## Figures and Tables

**Table 1:** Sociodemographic characteristics of the participants (n = 361).

Variables	n	%
Age (years)		
18	115	31.9
19–24	189	52.4
>24	57	15.7
Sex		
Female	210	58.2
Male	151	41.8
Discipline of studies		
Health Sciences	125	34.6
Other disciplines	236	65.4
Total	361	100

**Table 2:** Descriptive and comparative analysis of the variables under study according to the sex of the participants.

Variables	Male			Female			U *	*p* **
	Median	IQR (25–75)	Range (Min-Max)	Median	IQR (25–75)	Range (Min-Max)		
Sensory appeal	8.00	6.00–11.00	3.00–21.00	8.00	6.00–11.00	3.00–18.00	17,204.50	0.505
Health consciousness	8.00	6.00–10.00	3.00–21.00	8.00	6.00–10.00	3.00–17.00	16,009.50	0.073
Perception of FOP food labels	21.00	17.00–24.00	8.00–42.00	20.00	16.00–24.00	6.00–33.00	16,009.50	0.074
Purchase intention for unhealthy food	15.00	11.00–18.00	4.00–28.00	16.00	11.00–20.00	4.00–28.00	16,935.00	0.358

* The Mann-Whitney U test was used; FOP = Front-of-pack food labels; ** *p*-value.

**Table 3:** Spearman correlations between the study variables.

Variables	Sensory Appeal	Health Consciousness	Perception of FOP Food Labels	Purchase Intention for Unhealthy Food
Sensory appeal	1	−0.080	0.069	0.346 **
Health consciousness	−0.080	1	0.200 **	−0.261 **
Perception of FOP food labels	0.069	0.200 **	1	−0.221 **
Purchase intention for unhealthy food	0.346 **	−0.261 **	0.221 **	1

The Spearman test was used. ** *p* < 0.01 (bilateral). FOP = Front-of-pack food labels.

**Table 4:** Multiple regression coefficients.

Model 1	Unstandardized Coefficients		Standardized Coefficients		
	B	SE	β	t	*p*
(Intercept)	10.283	1.221	-	8.425	<0.001
health consciousness	−0.529	0.083	−0.296	−6.389	<0.001
Sensory appeal	0.515	0.069	0.339	7.408	<0.001
Perception of FOP food labels	−0.238	0.047	−0.237	−5.114	<0.001
Sex	−0.320	0.070	−0.190	−4.571	<0.001
Age	−0.089	0.062	−0.054	−1.435	0.152
Discipline of studies	−0.410	0.081	−0.250	−5.062	<0.001

Note. Predictors: (constant) Health consciousness, sensory appeal, perception of FOP food labels; Dependent variable: Purchase intention for unhealthy food. FOP = Front-of-pack food labels. B = Unstandardized regression coefficient, SE = Standard error, β = Standardized regression coefficient, t = t-value, *p* = *p*-value.

## Data Availability

The data presented in this study are available on request from the corresponding author. The data are not publicly available due to privacy and ethical concerns.

## Supplementary Materials

The following supporting information can be downloaded at: https://www.mdpi.com/article/10.3390/nu17111921/s1: Questionnaire.

## Abbreviations

The following abbreviations are used in this manuscript:

FOP	front-of-package
