# Supplementary material for: Health Consciousness, Sensory Appeal, and Perception of Front-of-Package Food Labels as Predictors of Purchase Intention for Unhealthy Foods in Peruvian University Students
Source: Nutrients. 2025 Jun 3;17(11):1921. doi: 10.3390/nu17111921 (PMC12156990; doi:10.3390/nu17111921)
Supplement: Supplementary file 1 [file nutrients-17-01921-s001.zip › nutrients-3650889-Supplementary Materials.pdf]

Questionnaire

Purpose of the Questionnaire

This questionnaire aims to collect information regarding the influence of health consciousness, sensory appeal, and front-of-package labeling on the purchase intention of unhealthy foods among university students. Your responses will help understand consumer behavior and decision-making regarding food choices.

Instructions

- Your participation is voluntary, and your responses will remain anonymous and confidential.
- Please read each question carefully before selecting your answer.
- Mark the option that best represents your opinion or experience with an "X".
- There are no right or wrong answers; we are only interested in your honest opinions.
- If you have any questions or concerns, please ask the researcher before proceeding.

Your input is greatly appreciated. Thank you for your time and participation!

Sensory Appeal

1. Unhealthy food looks appealing.
   a. ___Strongly agree (7)
   b. ___Agree (6)
   c. ___Slightly agree (5)
   d. ___Neutral (4)
   e. ___Slightly disagree (3)
   f. ___Disagree (2)
   g. ___Strongly disagree (1)

2. Unhealthy foods have a pleasant texture.
   a. ___Strongly agree (7)
   b. ___Agree (6)
   c. ___Slightly agree (5)
   d. ___Neutral (4)
   e. ___Slightly disagree (3)
   f. ___Disagree (2)
   g. ___Strongly disagree (1)

3. Unhealthy food tastes good.
   a. ___Strongly agree (7)
   b. ___Agree (6)

   c. ___Slightly agree (5)
   d. ___Neutral (4)
   e. ___Slightly disagree (3)
   f. ___Disagree (2)
   g. ___Strongly disagree (1)

Health Consciousness

4. I carefully choose food to ensure good health.
   a. ___Strongly agree (7)
   b. ___Agree (6)
   c. ___Slightly agree (5)
   d. ___Neutral (4)
   e. ___Slightly disagree (3)
   f. ___Disagree (2)
   g. ___Strongly disagree (1)

5. I consider myself a health-conscious consumer.
   a. ___Strongly agree (7)
   b. ___Agree (6)
   c. ___Slightly agree (5)
   d. ___Neutral (4)
   e. ___Slightly disagree (3)
   f. ___Disagree (2)
   g. ___Strongly disagree (1)

6. I often think about health-related matters.
   a. ___Strongly agree (7)
   b. ___Agree (6)
   c. ___Slightly agree (5)
   d. ___Neutral (4)
   e. ___Slightly disagree (3)
   f. ___Disagree (2)
   g. ___Strongly disagree (1)

Perception of Front-of-Package Labeling

7. The warning label on food packaging influences my food choices.
   a. ___Strongly agree (7)
   b. ___Agree (6)
   c. ___Slightly agree (5)
   d. ___Neutral (4)
   e. ___Slightly disagree (3)
   f. ___Disagree (2)
   g. ___Strongly disagree (1)

8. Prices and promotions influence my purchases.
   a. ___Strongly agree (7)
   b. ___Agree (6)
   c. ___Slightly agree (5)
   d. ___Neutral (4)
   e. ___Slightly disagree (3)
   f. ___Disagree (2)
   g. ___Strongly disagree (1)
9. I would like to follow the warning labels' recommendations, but I do not have time to read them.
   a. ___Strongly agree (7)
   b. ___Agree (6)
   c. ___Slightly agree (5)
   d. ___Neutral (4)
   e. ___Slightly disagree (3)
   f. ___Disagree (2)
   g. ___Strongly disagree (1)
10. I would like to follow the warning labels' recommendations, but I do not know how to interpret them.
   a. ___Strongly agree (7)
   b. ___Agree (6)
   c. ___Slightly agree (5)
   d. ___Neutral (4)
   e. ___Slightly disagree (3)
   f. ___Disagree (2)
   g. ___Strongly disagree (1)
11. I would like to follow the warning labels' recommendations, but I lack guidance on how to choose correctly.
   a. ___Strongly agree (7)
   b. ___Agree (6)
   c. ___Slightly agree (5)
   d. ___Neutral (4)
   e. ___Slightly disagree (3)
   f. ___Disagree (2)
   g. ___Strongly disagree (1)
12. I am satisfied with the implementation of the warning labels in Peru.
   a. ___Strongly agree (7)
   b. ___Agree (6)
   c. ___Slightly agree (5)
   d. ___Neutral (4)
   e. ___Slightly disagree (3)

f. ___Disagree (2)
g. ___Strongly disagree (1)

13. I am satisfied with the design and layout of the warning labels.
    a. ___Strongly agree (7)
    b. ___Agree (6)
    c. ___Slightly agree (5)
    d. ___Neutral (4)
    e. ___Slightly disagree (3)
    f. ___Disagree (2)
    g. ___Strongly disagree (1)

Unhealthy Food Purchase Intention

14. I am willing to consume unhealthy foods (e.g., sugary drinks, processed snacks, fast food) if they are available for purchase.
    a. ___Strongly agree (7)
    b. ___Agree (6)
    c. ___Slightly agree (5)
    d. ___Neutral (4)
    e. ___Slightly disagree (3)
    f. ___Disagree (2)
    g. ___Strongly disagree (1)

15. I intend to consume unhealthy foods (e.g., sugary drinks, processed snacks, fast food) if they are available for purchase.
    a. ___Strongly agree (7)
    b. ___Agree (6)
    c. ___Slightly agree (5)
    d. ___Neutral (4)
    e. ___Slightly disagree (3)
    f. ___Disagree (2)
    g. ___Strongly disagree (1)

16. I plan to consume unhealthy foods (e.g., sugary drinks, processed snacks, fast food) if they are available for purchase.
    a. ___Strongly agree (7)
    b. ___Agree (6)
    c. ___Slightly agree (5)
    d. ___Neutral (4)
    e. ___Slightly disagree (3)
    f. ___Disagree (2)
    g. ___Strongly disagree (1)

17. I will try to consume unhealthy foods (e.g., sugary drinks, processed snacks, fast food) if they are available for purchase.
    a. ___Strongly agree (7)

b. ___Agree (6)
c. ___Slightly agree (5)
d. ___Neutral (4)
e. ___Slightly disagree (3)
f. ___Disagree (2)
g. ___Strongly disagree (1)

SOCIODEMOGRAPHIC DATA

18. Age
    ____ (years)

19. Sex
    ___ Male
    ___ Female

20. Field of Study
    ___ Law/Political Science
    ___ Health Sciences
    ___ Psychology
    ___ Education
    ___ Administrative Sciences
    ___ Engineering/Architecture
    ___ Other: (Specify)
